# Supplementary material for: HIV viral load assays when used with whole blood perform well as a diagnostic assay for infants
Source: PLoS One. 2022 Jun 30;17(6):e0268127. doi: 10.1371/journal.pone.0268127 (PMC9246233; doi:10.1371/journal.pone.0268127)
Supplement: Attachment — Submitted filename: PLoS One Uganda VL for EID reviewer comments.docx [file pone.0268127.s001.docx]

Author responses to deputy editor and reviewer comments

We note that you have provided funding information that is not currently declared in your Funding Statement. However, funding information should not appear in the Acknowledgments section or other areas of your manuscript. We will only publish funding information present in the Funding Statement section of the online submission form.

Please remove any funding-related text from the manuscript and let us know how you would like to update your Funding Statement. Currently, your Funding Statement reads as follows:

 [The funders had no role in study design, data collection and analysis, decision to publish, or preparation of the manuscript.]

**Response**: we appreciate this information and have adjusted accordingly: removed from the Acknowledgements and included instead in the Funding Statement.

Additional Editor Comments (if provided):

Dear Authors,

Both reviewers are positive on your analysis and manuscript and recommend a revision. I basically agree with their recommendations and think their comments are important for you to address carefully. As a result, I have recommended a "major" revision to emphasize to you to respond carefully to their comments.

In addition, please be careful about making conclusions that are not directly supported by your analysis. For example, in the abstract conclusions, your conclusion about supply chain logistics, greater access, etc., is not supported by anything in your analysis.

**Response**: we appreciate this suggestion and have revised on page 2, lines 46-47.

You also provide no data to suggest that "fragmented and challenging laboratory systems" are the a major problem with early infant diagnosis.

**Response**: as we weren’t able to include references in the abstract, we have discussed and referenced this in the introduction on page 3, lines 59-62.

Please carefully review your presentation to make sure you have statements referenced when needed (e.g., see sentence ending line 55).

**Response**: we have now referenced that line as well as others as suggested and necessary.

Line 61: References 4 and 5 are fairly old. While such problem may persist in Uganda, Kenya and South Africa seem to be well organized for EID if mothers/infants return and if a sample is obtained at the clinic (or elsewhere).

**Response**: While Uganda, Kenya and South Africa might be leaders in EID, yearly data has still showed stagnation or stable coverage rather than increases. Further, this is also seen when reviewing ART initiation rates. Globally rates still remain unacceptably lower than adults. Many suggest that this is in part because even good lab-based EID systems may never be good enough. An upcoming EID systematic review as well as several studies looking at point-of-care with the comparator being lab-based testing, even in good EID systems, show such deficiencies and gaps. Even in those settings, solutions are needed. See here for reference: <https://www.who.int/news/item/17-03-2021-who-publishes-new-clinical-recommendations-on-hiv-prevention-infant-diagnosis-antiretroviral-therapy-initiation-and-monitoring>.

Line 63-65 seems to need a reference.

**Response**: Reference #5 added on page 3, line 65.

Line 84-85: This is the point of your paper, which is very reasonable. Once the answer to "can" is complete, then other analyses will be need to address if VL quantitative assays "should" be used (based on a variety of criteria including budget impact, cost-effectiveness, etc). You cannot address 'should' in this paper as far as I can tell.

**Response**: we agree, thank you, and will ensure this message is clearer in the discussion and conclusions.

And last, the data availability statement is not clear: "all relevant data are within the manuscript and its supporting information files". I see no SI file. Am I missing something?

Response: thank you for noting this. We have adjusted accordingly.

Reviewer #1: Major comment
The impact of changing the viral load assay for diagnosis of HIV in infants is highly significant and will have far-reaching effects. For example, it will require changing of individual country guidelines for use of antiretroviral drugs for treating and preventing HIV. Additionally, countries are currently moving from using Dried Blood Spots for viral load monitoring to plasma, therefore such a move will delay countries from this implementation. Lastly, the level of detection when DBS is used for viral load quantification is 839copies/ml. When this assay is used for Early Infant Diagnosis, the limit of detection in unclear.

**Response**: we have added a paragraph to the discussion on page 14, lines 261-272 as well as a sentence around defining the limits of determine and indeterminate ranges if this technique would be employed on page 15, lines 297-298.

Procurement issues have been cited as the reason for the change of assay. It is not clear why a procurement problem should be solved using a technical method. Therefore, the justification for using a viral load assay for diagnosis of HIV in infants is unclear to the reviewer.

**Response**: we have tried to clarify this in the introduction on page 3, lines 65-72.

Minor comments
Background
1. Line 76 should read ……when serological assays cannot be reliably used. Instead of….. when serological assays can be reliably used.

**Response**: this has been changed on page 4, line 81.

Methods
1. Line 88-89 state that all testing occurred at the Central Public Health Laboratory in Kampala, Uganda using routinely collected dried blood spot samples. Line 93-95 state that samples were purposefully selected in that all consecutively collected positive samples and an equal number of randomly selected negative samples were included and blindly tested each week until the target sample size was met. Did the study use remnant samples from routinely collected dried blood spot samples? Kindly clarify.

**Response**: the reviewer is correct. We have adjusted on page 5, line 95.

2. On which test platform where the samples first (initially) tested to allow for purposeful selection of positive samples and an equal number of negative samples for inclusion to the study?

**Response**: we have tried to clarify this and added to page 5, lines 99-102.

3. The method section of the abstract states that infants for testing were enrolled using either the Roche or Abbott molecular technologies. Yet, this in not mentioned in the text. Kindly clarify.

**Response**: we tried to clarify this in the abstract on page 2, line 33 as well as in the methods on page 5, lines 99-102.

4. The methods section of the abstract states that dried blood spot samples were processed for both assays on the Abbott using the manufacturer’s standard early infant diagnosis protocol. However, only testing on the Abbott RealTime HIV-1 viral load assays is mentioned in the text. Kindly clarify.

**Response**: we have tried to clarify this within the methods in several sections: page 5, lines 99-102 and 109-100 as well as page 6, lines 140-142.

5. The methods section of the abstract states that all infants received a qualitative test for clinical management and additional paired quantitative tests. Yet, the text mentions that Viral load test results were not provided to patients. The routine clinical qualitative early infant diagnosis test results were returned to the health care facility and caregiver per national guidelines. Kindly clarify.

**Response**: that is correct and current national guideline practice. Providing the viral load result is not in line with national clinical guidelines and thus were not provided. This is reiterated on page 7, lines 150-152.

6. Lines 99-100 state that… “…Separate sets of consecutively collected positive samples were used for the two technologies (Roche and Abbott)…...”Kindly specify the assays used for testing the positive sample in the two test platforms.

**Response**: this has been added on page 5, lines 109-110.

Reviewer #2: Thank you for your manuscript. This study adds a valuable contribution to the options for increasing EID coverage. The methods and results and interpretation are clear and sound.

Line 33 _ Methods in the abstract - would be helpful to mention Uganda as the setting.

**Response**: this has been added on page 2, lines 33 and 34-35.

In the introduction there is no mention of the option of POC testing for EID and the superior clinical utility from this testing as evidenced in the EGPAF-UNITAID rollout in 9 African countries. <https://www.pedaids.org/impact/data-dashboard/point-care-early-infant-diagnosis-data-dashboard/>
Even though this is a laboratory evaluation, POC testing should be mentioned as an alternative EID strategy in the introduction to increase testing.

**Response**: while this isn’t super relevant to address the issues at hand, we have added the option of POC as a solution in the introduction, including appropriate references, on page 3, lines 67-68.

Line 77 - when serological assays "cannot' be reliably used - this would make more sense as antibody testing is not accurate <18months.

**Response**: thank you for catching this, we have adjusted on page 4, line 81.

Line 149 - would it not be more accurate to say - 68% "had been on some form of prophylaxis" as only 33% of infants were <3months and yet 58% report Daily NVP to 6 weeks suggesting it is not current use?

**Response**: we have adjusted on page 8, line 158.

Line 55 - The 18, 3 and 36 invalid or depleted samples are not accounted for in the tables 3 and 4. It would help to see how these were handled and differentiate between the invalid and depleted.

**Response**: these were not included in the analysis as they did not provide a valid result nor had additional sample to retest. We added this clarification on page 9, lines 165-166.

Line 226 - This comment that '75% indeterminate test results are negative' is not stated in the referenced article. Another article " Interpretation of indeterminate HIV-1 PCR results are influenced by changing vertical transmission prevention regimens. by Maritz J, Maharaj JN, Cotton MF, Preiser W. in the Journal of Clinical Virololgy. 2017;95:86–9 reports that the positivity rate of indeterminate results had increased to 64% in South Africa with Option B+ which contradict your statement. Indeterminate results and undetectable VL results continue to be a challenge in the era of infant prophylaxis and maternal ART.

**Response**: we changed the reference to the most up-to-date, 2018 WHO guidelines that performed a systematic review and meta-analysis on this issue. See page 13, line 237.

Line 273 - The phrase 'if desired' is not necessary as a viral load result is always clinically useful as a baseline from which to monitor treatment.

**Response**: we deleted that phrase accordingly.

Line 286 - It would be better to say "generalizable to other LMIC settings"

**Response**: we have revised accordingly on page 16, line 309.

Line 287 - more accurate to say most infants "received some" prophylaxis

**Response**: we have done so on page 16, line 301.

Line 292 - These are not high rates of treatment exposure - they are both suboptimal for both maternal ART and infant prophylaxis. Agree that confirmatory testing most important.

**Response**: agreed, we have revised on page 16, line 311 and 317.

Line 303 - Generalizable in similar settings.

**Response**: agreed, we revised on page 17, line 327.
